# Supplementary material for: Methods for constructing and evaluating consensus genomic interval sets
Source: Nucleic Acids Res. 2024 Aug 24;52(17):10119–31. doi: 10.1093/nar/gkae685 (PMC11417377; doi:10.1093/nar/gkae685)
Supplement: gkae685_Supplemental_File [file gkae685_supplemental_file.pdf]

Supplementary Methods

Building input coverage tracks

The input to our universe-building methods is genome signal tracks that count the sum of start positions, end positions, and overlaps at each base. To do this, we developed a fast algorithm that allows us to produce all three of these tracks using a single pass through the reads.

First, each region set in the collection is sorted by sequence name (chromosome) and start position. Then, these region sets are merged into a single, sorted region set that contains all regions in the collection; this combined region set now contains potentially many overlapping regions. We process each chromosome independently. For each chromosome, we first sort the ends separately. This decouples the interval pairs, so they no longer represent regions; for our use cases, we don't require that pairing, and this step allows us to process the files in a single pass, making the computation very fast.

We initialize a coverage variable to 0, which will indicate the coverage for the current position in the genome. To make signal tracks more stable, for start and end positions, we smooth the tracks by a smoothing window size w. To do this, we adjust the boundary position by subtracting $w/2$, and push to a stack of "smoothed window ends" the value of $p + w/2$. This effectively turns the start and end position into a region; for starts we have their start and end values, for ends we have their start and end values. They thus can use the same algorithm we use for the coverage tracks, which simply use the original (non-smoothed) start and end positions.

Given a list of sorted starts and ends, we use a triple plane-sweep algorithm to go through the positions in the genome and the sorted starts and ends using nested loops. In the outer loop, we loop through bases in the genome; in the inner loop, through regions. At each position in the genome, we increment $coverage$ variable for region starts at this position, and decrement the variable for each interval that ends. We then we emit the current number of overlapping regions. This yields a base-pair-level signal track. The same algorithm can be used for smoothed start, smoothed end, or raw coverage signal tracks.

Dealing with noise through smoothing

The point of this smoothing step is to deal with noise in the region boundaries. Experimental sets of regions, like most measurements, contain noise. Addressing this noise is a core part of what the models we are building is to do. An example of a consensus algorithm that does not consider noise is the simple union approach, which will pick up all regions that show up in any input region set. The goal of the more sophisticated models is to reduce noise, without being too aggressive and eliminating true signal. Smoothing deals with noise by accommodating randomness in the exact placement of a peak start and end. Before we feed our observed tracks into the HMM, we smooth the peak boundaries (by a customizable parameter). This has the effect of canceling out variation due to noise in starting location. For practical purposes, the starting position of a peak is often not important down to a specific single nucleotide; hence, smoothing out across a range of 10, 25, or 50 bases allows the model to more accurately hone in on the most likely start and end positions. The level of smoothing can be tuned depending on the level of noise in the input data.

Coverage cutoff universe

Probabilistic models of coverage and background

To improve our description of how a genome is covered by collection of files, we propose a probabilistic approach. Given a collection of n region sets $\mathbb{R} = [\mathcal{R}_1, \mathcal{R}_2, ...\mathcal{R}_n]$ where $\mathcal{R}_i$ denotes a region set $\mathcal{R}_i = [r_1, r_2, ...r_m]$, and r_i denotes a single region in the set, we make a model describing the probability of each position in the genome being covered by a region in a randomly selected region set from the collection. To compute this probability, we first compute the coverage frequency for each genomic position independently, $freq_c$, a vector of length g (the length of the genome). We do this by simply counting, for each position, in how many files it is covered, yielding frequencies of coverage for each genomic position. We then convert $freq_c$ into a probability distribution over the genome by dividing each element by the sum. We define the probability of a position being covered as:

$$\pi_i^c = \frac{freq_c(i)}{S_c} \tag{1}$$

where i indicates the position (base-pair) and S_c denotes sum of coverage frequency counts across the whole genome:

$$S_c = \sum_{i=1}^{g} freq_c(i)$$

This variable, π_i^c, can be thought of as an extension of a multinomial random variable. Consider a multinomial random variable, where the categories are positions (individual base pairs) in the genome. We seek to define a distribution across these positions. However, our model does not follow a simple sampling model either with replacement or without replacement; instead, we employ a hierarchical sampling model, where samples (base pairs) are first grouped (representing files), where groups may not duplicate items, indicating a without replacement sampling strategy. But across groups, duplicates are allowed, indicating a with replacement sampling strategy. Thus, π_i^c represents a probability distribution across the genome of whether a base is

covered in the region set collection $\mathbb{R}$. In other words, if we think of drawing random bases from a set of files, this distribution describes the probability of selecting each base, using our hierarchical sampling strategy.

Next, we calculate an independent probability distribution for the inverse model, the probability of each position being *not covered* in our sample data, which we call background:

$$freq_{c'}(i) = n - freq_c(i), \tag{2}$$

where i again represents genome position and n is the number of files. As before, we divide these frequencies by their sum to get a probability distribution across the genome, this time of background probabilities:

$$\pi_i^{c'} = \frac{freq_{c'}(i)}{S_{c'}}, \tag{3}$$

where $S_{c'}$ denotes sum of genome background (the total number of bases that were not covered in a set of files):

$$S_{c'} = \sum_{i=1}^{g} freq_{c'}(i) = gn - \sum_{i=1}^{g} freq_c(i). \tag{4}$$

Where g is the length of the genome, Thus, gn represents the maximum possible elements that could be covered in a given collection of n files; the unit could be “file-bases”.

Combing information from those two probabilities, $\pi_{c'}$ and π_c, we get a model M describing for each position how probable it is for it to be covered (core) or not (background).

$$M(i) = [\pi_i^{c'}, \pi_i^{c}] \tag{5}$$

These two probability distributions are not entirely independent, but they are also not exact inverses because of the complicated file structure, which affects the sampling strategy.

Finding likelihood cut-off value

We can use presented model to find the most probable universe (under the model). To do it, we have identify all the bases for which the probability of being covered is higher than the probability of being not covered; that is, those that satisfy the following inequality:

$$\pi_i^{c'} \leq \pi_i^{c}.$$

Substituting the definitions from (1) and (3), this is equivalent to:

$$\frac{freq_{c'}(i)}{S_{c'}} \leq \frac{freq_c(i)}{S_c}.$$

Then, using equations (2) and (4), we get:

$$\frac{n - freq_c(i)}{gn - S_c} \leq \frac{freq_c(i)}{S_c}.$$

Multiply both sides by $S_c(gn - S_c)$:

$$nS_c - S_c freq_c(i) \leq gn freq_c(i) - S_c freq_c(i).$$

Simplifying:

$$nS_c \leq gn freq_c(i).$$

Solving this equation for $freq_c(i)$, results in:

$$freq_c(i) \geq \frac{S_c}{g}.$$

Thus, if we have identified a cut-off value ($\frac{S_c}{g}$); we take the maximum likelihood universe under this model for a given collection as the one for which any locations $freq_c(i)$ above the cutoff are included, and others are excluded.

Maximum likelihood universe

Probabilistic model extended to region boundaries

The previous probabilistic description a region set collection across genome considers only region coverage; it does not include information about the position of regions' boundaries. To extend the model, we make similar models for start and end boundary probabilities. We calculate the frequencies of each position containing either start $freq_s$ or end $freq_e$. Moreover, as before, for each base pair, we count the number of region sets in which it is background (in other words, *not* a start), for start as $freq_{s'} = n - freq_s$, and for end (*not* an end) as $freq_{e'} = n - freq_e$. Using that, we derive for each position the probability of it being a boundary or boundary's background:

$$\pi_i^s = \frac{freq_s(i)}{S_s} \quad \text{where} \quad S_s = \sum_{i=1}^{g} freq_s(i)$$

$$\pi_i^{s'} = \frac{freq_{s'}(i)}{S_{sb}} \quad \text{where} \quad S_{s'} = \sum_{i=1}^{g} freq_{s'}(i)$$

$$\pi_i^e = \frac{freq_e(i)}{S_e} \quad \text{where} \quad S_e = \sum_{i=1}^{g} freq_e(i)$$

$$\pi_i^{e'} = \frac{freq_{e'}(i)}{S_{e'}} \quad \text{where} \quad S_{e'} = \sum_{i=1}^{g} freq_{e'}(i)$$

Thus, π_i^s is the probability of being a start, $\pi_i^{s'}$ is the probabilty of *not* being a start, π_i^e is the probability of being an end, and $\pi_i^{e'}$ is the probabilty of *not* being an end.

Combining these probabilities with the model described in (5) we get a complex model describing the probability distribution of a collection of files across the genome:

$$M_{\text{complex}}(i) = \begin{bmatrix} \pi_i^{s'} & \pi_i^s & \pi_i^{c'} & \pi_i^c & \pi_i^{e'} & \pi_i^e \end{bmatrix}. \quad (6)$$

Building maximum likelihood universe

We can use this extended likelihood model (6) to derive a maximum likelihood flexible universe. We do it by finding an optimal genome segmentation with four states: start s, core c, end e, and background b. These states reflect the parts of a flexible region. We assume that this can be found by dividing the problem into overlapping sub-problems. That means that the best segmentation of first i positions is a result of the most likely segmentation of first $i-1$ positions and the most likely state at position i. This assumption makes the problem amenable to dynamic programming, which we employ to find the maximum likelihood universe.

Next, we need to create a scoring function that considers all the probabilities described earlier. We reason that a score for assigning a particular base to a particular state could be computed by multiplying the probability of the state with the probabilities of *not* being in the other states. For example, we compute the probability of being in state s as $\pi_i^s \pi_i^{c'} \pi_i^{e'}$, and equivalently for state c and state e. To compute the probability of being in none of these states, or, equivalently, in the overall background state b, we can compute $\pi_i^{s'} \pi_i^{c'} \pi_i^{e'}$ (multiplying all three background probabilities). Using this approach, we create a scoring function P that for each position in the genome i denotes its probability of being in a given state:

$$P(i|state = s) = \pi_i^s \pi_i^{c'} \pi_i^{e'}, \quad P(i|state = c) = \pi_i^c \pi_i^{s'} \pi_i^{e'}, \quad P(i|state = e) = \pi_i^e \pi_i^{s'} \pi_i^{c'}, \quad P(i|state = b) = \pi_i^{s'} \pi_i^{c'} \pi_i^{e'}.$$

Using P, we can represent our problem as a recursive equation:

$$F(i, state) = P(i, state) + max(F(i-1, state_{prev}), F(i-1, state)),$$

$$F(0, state) = P(0, state)$$

where i denotes position in the genome, $state \in \{s, c, e, b\}$ is the current state, and $state_{\text{prev}}$ a state from which $state$ can be accessed, for example if $state = s$ than $state_{\text{prev}} \in \{s, b\}$. Using this equation, we use dynamic programming to find a most likely path.

LH universe filtering

Although the LH universe is designed to find an optimal universe, it can result in very small universe regions. We remedy that by removing small regions (below 100 bp). Furthermore, for flexible likelihood universes, we also add a filtering step; for each region we calculate its contribution to the flexible universe likelihood by calculating the difference between the total universe likelihood and the likelihood with the current region removed (Fig. S2). This *flexible universe likelihood* score is based on a different conceptualization of the region likelihoods (see section on universe likelihood in Methods), and thus, individual regions may contribute negatively to that score. Based on that, we filter out regions with negative contribution to the universe likelihood.

Hidden Markov Model universe

Process of determining HMM parameters

One advantage of the HMM is that we have control over parameters for the transition and emission probabilities, allowing us to tweak the model. In many applications of HMMs, these parameters are trained using gold-standard reference input data and the forward-backward algorithm. However, in our case, we lack suitable training data. We attempted to construct reference datasets that could be used to train parameters, but were unsatisfied with the final trained models, which would often tend to converge to parameter sets that yielded no universe regions. Instead, we decided to parameterize the HMM manually by tuning the system to yield the type of results we sought. We found that, through trial and error, we were able to achieve better results.

In the process of tuning the HMM and applying it to different sizes of input region set collections, we realized that the parameters would need to vary depending on the input dataset. Since we desired a universal model that could be applied in many different scenarios, we reasoned that a model based on a pre-processed normalized signal could be made to work across varying input scenarios. Therefore, we introduced a pre-processing step of quantile normalization of the input signal tracks. For each track, we map its values to a reference distribution, in a way that normalizes the distribution to have the same quantile values as the reference distribution. As a reference, we use Negative Binomial distribution, since in most cases it represents genome coverage by the collection, with different success probabilities for cores and boundaries, respectively 0.2 and 0.1, and same number of successes, which is equal to 1. Then, we feed the normalized signals into the HMM. This approach allows us to use a single set of emission probabilities for diverse collection sizes, thereby enabling the HMM to process diverse data collections without requiring collection-specific tuning.

This quantile normalization step allows us to handle different sizes of input collection, making it more universally useful; however, it may still be desirable to tune the model differently depending on the noise of the input collection. Tuning the emission and transition probabilities also allows us to dial the sensitivity and specificity of the model, which provides the ability to handle data with different single-to-noise ratios. For example, by allowing the background state to emit higher start, coverage, and end signal, the model will be less likely to create universe regions where a small minority of input sets are covered, and vice versa. The final model we used is based on one empirical evaluation of universes and attempt to strike a practical balance, but interested users could tweak the parameters to dial up or down the noise tolerance.

Parameters

Since it is very unlikely for the process to start in something different from background, we assign to it the highest probability p_{start}. In transition matrix T, we define the peak structure by setting to zero impossible transitions. Moreover, we set a probability of staying in a given state much higher than probability of transiting to the next. This way, we can make sure that segments are not too short. The third matrix is an emission matrix E, which describes probability of given values being emitted from a given state. We used a Poisson distribution with manually tuned parameter λ for each emitted variable (starts, coverage, and ends).

$$p_{\text{start}} = \begin{bmatrix} 0.01 & 0.01 & 0.97 & 0.01 \end{bmatrix}, \tag{2}$$

$$T = \begin{bmatrix} 1 - 10^{-11} & 10^{-11} & 0 & 0 \\ 0 & 1 - 10^{-7} & 10^{-7} & 0 \\ 0 & 0 & 1 - 10^{-11} & 10^{-11} \\ 0.1 & 0 & 0 & 0.9 \end{bmatrix}, \tag{3}$$

$$E = \begin{bmatrix} 5 & 3 & 0.0001 \\ 0.05 & 5 & 0.05 \\ 0.0001 & 3 & 5 \\ 10^{-5} & 10^{-4} & 10^{-5} \end{bmatrix} \tag{4}$$

In matrix T, the rows and columns correspond to the four states in this order: start, coverage, end, background.

In matrix E, the rows are the same: start, coverage, end, and background, and the columns correspond to the 3 observed emission variables: start, coverage, and end.

HMM universe filtering

We remove from the raw HMM universe small regions below 100 bp. Additionally, as for the LH universe, we filter regions, which have strong negative contribution to the universe likelihood score (<-100) (Fig. S3).

Calculating flexible universe likelihood given model

Overview

We seek to compute a likelihood score for a proposed universe given a collection of region sets. We previously described a likelihood model used to *create* a universe given a collection (which we called the likelihood universe). At first, we tried to apply the original model as a universe assessment method; however, this likelihood model could not be used on flexible universes because boundaries, which are now regions instead of points, contribute very negatively to the score, since the probabilities of emitting a boundary are lower than coverage due to their sparsity. Therefore, we also derived a separate likelihood approach to be used for evaluating flexible universes.

Hard universe

First, we describe the simpler case of a hard (not flexible) universe, and we will then extend this to the case of a flexible universe. For hard universes, we *can* use the original likelihood to evaluate, because they don't have the problem introduced by flexible starts and ends. To calculate the likelihood of any universe given region set collection $\mathbb{R}$, we first create a binary matrix representation of the universe, U_{binary}. We build U_{binary} with 6 rows, corresponding to 1) start-background, 2) start, 3) core-background, 4) core, 5) end-background, and 6) end. The columns correspond to genome positions. We set the value to one if, at the given position, the universe contains the corresponding part of the peak.

For example, for a region r with start $s = 3$ and, end $e = 7$ we represent part of the genome as:

$$U_{binary} = \begin{bmatrix} s'_i \\ s_i \\ c'_i \\ c_i \\ e'_i \\ e_i \end{bmatrix} = \begin{bmatrix} \dots & 1 & 1 & 0 & 1 & 1 & 1 & 1 & \dots \\ \dots & 0 & 0 & 1 & 0 & 0 & 0 & 0 & \dots \\ \dots & 1 & 1 & 0 & 0 & 0 & 0 & 1 & \dots \\ \dots & 0 & 0 & 1 & 1 & 1 & 1 & 0 & \dots \\ \dots & 1 & 1 & 1 & 1 & 1 & 0 & 1 & \dots \\ \dots & 0 & 0 & 0 & 0 & 0 & 1 & 0 & \dots \end{bmatrix}$$

Where i corresponds to genome position, yielding a long $6 \times n$ matrix, n is the length of the sequence.

Then, we can calculate the log-likelihood of the universe using a matrix multiplication:

$$\log\left(L_h(U)\right) = U_{binary}^T * \log(M_{\text{complex}}) \tag{5}$$

Where U^T indicates the transpose operation.

Flexible universe

The flexible universe is trickier because the original data have point boundaries, but the universe has flexible boundaries. Therefore, we cannot compute probabilities in the same way, using the frequency of boundaries in the region set collection.

To address this, we start with a similar idea to calculate likelihood of a flexible universe, but we model start and end not as points, but subregions with uniform probability of containing a given boundary. To accommodate that, we introduce weights in U_{binary} matrix representation. For example, for region r with $s_{\text{start}} = 5$, $s_{\text{end}} = 8$, $e_{\text{start}} = 15$, $e_{\text{end}} = 20$ we represent it as:

$$U_{flex} = \begin{bmatrix} s_i' \\ s_i \\ c_i' \\ c_i \\ e_i' \\ e_i \end{bmatrix} =$$

$$\begin{bmatrix} \dots & 1 & \frac{2}{3} & \frac{2}{3} & \frac{2}{3} & 1 & \dots & 1 & 1 & 1 & 1 & \dots \\ \dots & 0 & \frac{1}{3} & \frac{1}{3} & \frac{1}{3} & 0 & \dots & 0 & 0 & 0 & 0 & \dots \\ \dots & 1 & \frac{2}{3} & \frac{1}{3} & 0 & 0 & \dots & 0 & 0 & \frac{1}{5} & \frac{2}{5} & \dots \\ \dots & 0 & \frac{1}{3} & \frac{2}{3} & 1 & 1 & \dots & 1 & 1 & \frac{4}{5} & \frac{3}{5} & \dots \\ \dots & 1 & 1 & 1 & 1 & 1 & \dots & 1 & \frac{4}{5} & \frac{4}{5} & \frac{4}{5} & \dots \\ \dots & 0 & 0 & 0 & 0 & 0 & \dots & 0 & \frac{1}{5} & \frac{1}{5} & \frac{1}{5} & \dots \end{bmatrix}$$

Then, we simply use the same matrix multiplication to calculate the likelihood with this weighted matrix:

$$\log\left(L_h(U)\right) = U_{flex}^T * \log(M_{\text{complex}})$$

The intuition behind this approach is that the flexible region is not defining a specific point for the region boundary; therefore, the contribution of the likelihood from the boundary distribution should be distributed across the flexible component. For the start and end probabilities, we thus divide the weight evenly across the segment; the denominator thus equals the length of the segment. The corresponding background is set to 1 - the state weight. To accommodate this dispersion of probability score, we also adjust the weights of the core state, which increases as the genome progresses because if a start had happened in the preceding base, then the next base would increase in probability of being in a core region. In other words, within a start state, as you progress along the genome, the probability of being in a core should increase. To model this, we simply have the probabilities of being in a core start at zero at the leftmost boundary of the start state, and increase to 1 at the rightmost boundary of the start state. Then, we do the inverse at the end states, starting at 1 and ending at 0.

General comparison of universe statistics

Data-driven universes tend to cover a similar amount of the genome as the data used to build it, except for universes derived from likelihood models (CC, CCF, LH universes) for large collections (CTCF ChIP large and TF ChIP), where the coverage drops from around 90% for original data to below 50% for universe (Fig. S4). This observation is the consequence of Eq. 1, which states that as the sum of coverage of the genome by collection increases, so does the maximum likelihood cutoff value. That means that for collection with high sum of genome coverage by collections, many positions with low coverage will be excluded. Moreover, union universe produces large peaks, especially for ChIP-Seq collections (CTCF ChIP small, CTCF ChIP large, TF ChIP); for TF collection average peak size is over 40 500, where peak size in the data is around 400. Interestingly, for CTCF ChIP large both HMM and LH universe have many small regions, which reflects the complicated nature of the data.

Accounting for noise in universe evaluation

The evaluation metrics account for noise through their asymmetry. Many of the evaluation metrics we proposed are asymmetric versions of evaluation parameters; for example, the F_{10} score penalizes one direction 10 times more than the other. The point of the asymmetry is that for these models, it is more problematic to miss real regions than to have a few extra regions. This framework allows the level asymmetry to be tuned; so a user who needed a universe that is highly sensitive could dial the metrics in a way to penalize losing information more. This would create universes that have more peaks, but may have more noise. On the other hand, for an application in which it's important to filter out more noise, the evaluations could be tuned in the other direction.

Supplementary Tables

***Table S1*: Statistics of demo region set collections.** *Five collections of regions ranging from 40 to >8,000 files, giving a diverse set of possible use cases where generating a set of consensus regions could help with data integration.*

Collection	N files	Region number	Genome covered (%)	Average region size
CTCF ChIP small	40	7,107,842	42.5%	367
CTCF ChIP large	877	152,694,264	89%	358
TF ChIP	8,503	1,463,013,952	90.7%	394
B-LCL ATAC	400	776,673	0.2 %	268
Random ATAC	5,000	76,332,694	17.8%	290

***Table S2*: Statistics of universes.** *Basic statistics of all presented universes, including the number of regions in the universe, their average size, and the percentage of the genome they cover.*

Collection	Universe	Region number	Average region size	Genome covered (%)
CTCF ChIP small	union	2,320,577	566	40.9 %
CTCF ChIP large	union	151,846	18,116	85.7 %
TF ChIP	union	69,209	40569	87.5 %
B-LCL ATAC	union	14,101	357	0.2 %
Random ATAC	union	1,306,508	421	17.1 %
CTCF ChIP small	coverage	2,120,192	572	37.8 %
CTCF ChIP large	coverage	1,368,624	504	21.5 %
TF ChIP	coverage	957,516	1030	30.7 %
B-LCL ATAC	coverage	14,027	357	0.2 %
Random ATAC	coverage	547,740	387	6.6 %
CTCF ChIP small	CCF	1,509,661	605	28.4 %
CTCF ChIP large	CCF	492,846	961	14.8 %
TF ChIP	CCF	612,181	1639	31.3 %
B-LCL ATAC	CCF	9,835	413	0.1 %
Random ATAC	CCF	316,089	618	6.1 %
CTCF ChIP small	ML	3,381,856	264	27.8 %
CTCF ChIP large	ML	3,042,529	218	20.7 %
TF ChIP	ML	2,887,104	364	32.7 %
B-LCL ATAC	ML	15,538	353	0.2 %
Random ATAC	ML	694,194	382	8.3 %
CTCF ChIP small	HMM	4,950,358	271	41.8 %
CTCF ChIP large	HMM	10,618,927	194	64.4 %
TF ChIP	HMM	7,896,890	308	75.7 %
B-LCL ATAC	HMM	11,818	406	0.1 %
Random ATAC	HMM	1,292,045	338	13.6 %
predefined	tiles1000	2,938,211	1000	91.5 %
predefined	Regulatory Build	548,310	950	16.2 %
predefined	SCREEN	1,063,878	273	9.0 %

Supplementary Figures

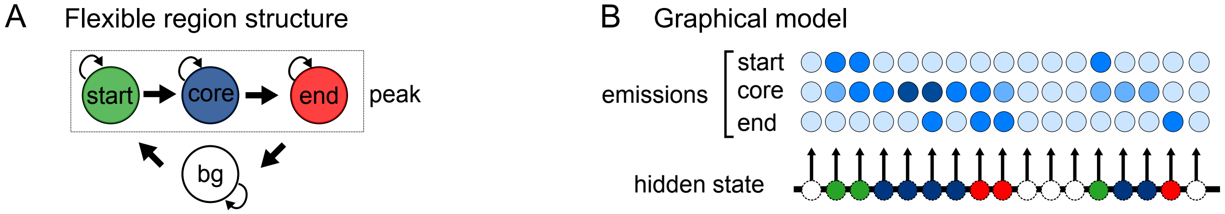

***Figure S1*: Details of Hidden Markov Model.** *A) State space diagram showing state transitions for the Hidden Markov Model. This is a graph representation of a flexible region. B) Graphical model describing the sequential states of the HMM.*

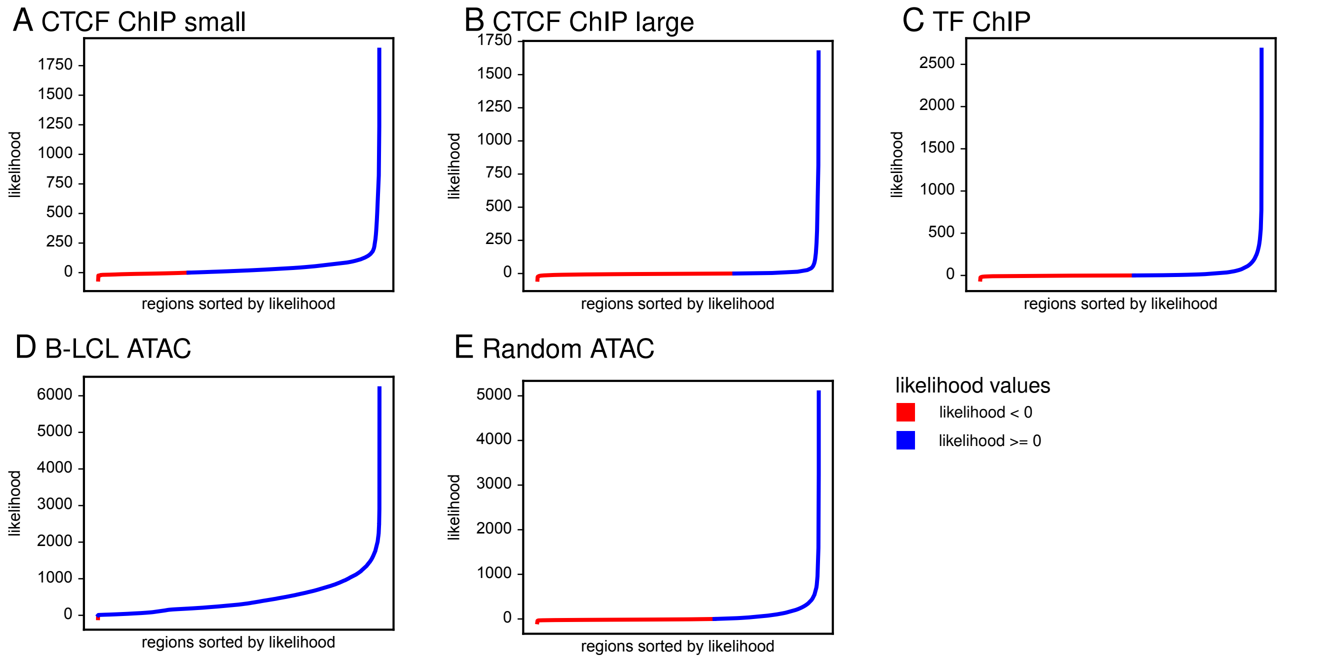

Figure* S2*: Sorted likelihood inputs of all regions present in the ML universe. *A) Likelihoods of regions in ML universe for CTCF ChIP small collection. B) Likelihoods of regions in ML universe for CTCF ChIP large collection. C) Likelihoods of regions in ML universe for TF ChIP collection. D) Likelihoods of regions in ML universe for B-LCL ATAC collection. E) Likelihoods of regions in ML universe for Random ATAC collection.*

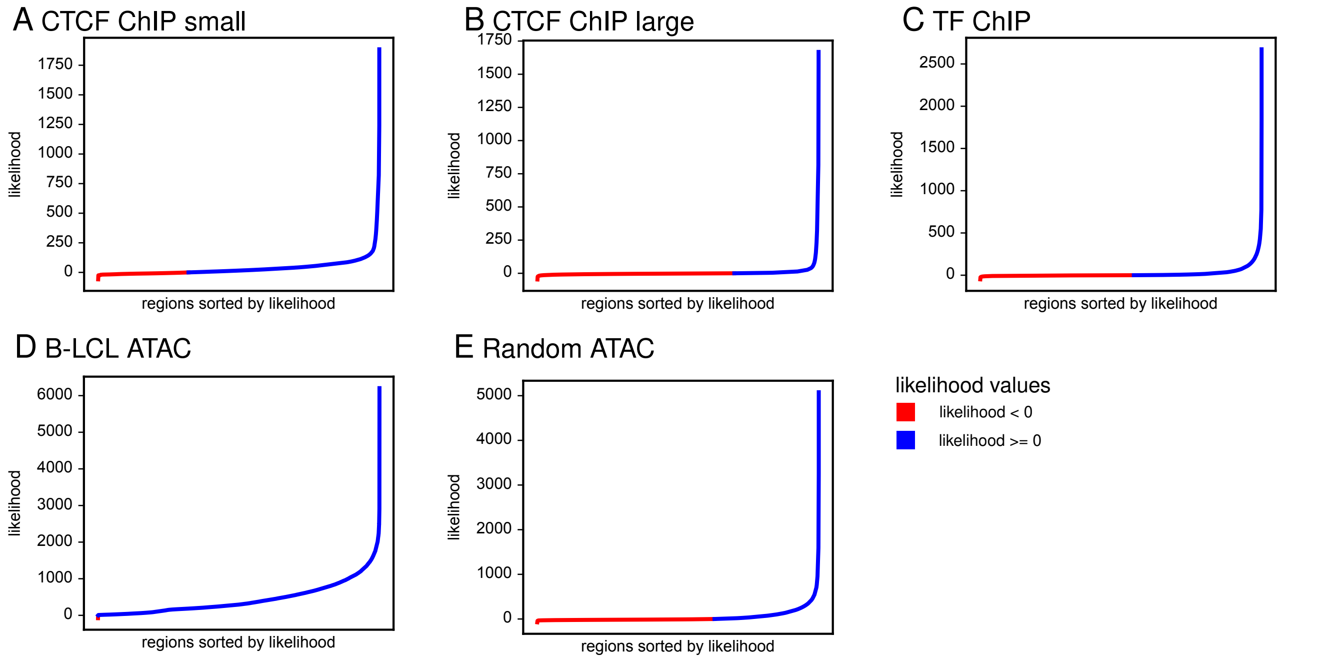

Figure* S3*: Sorted likelihood inputs of all regions present in the HMM universe. *A) Likelihoods of regions in HMM universe for CTCF ChIP small collection. B) Likelihoods of regions in HMM universe for CTCF ChIP large collection. C) Likelihoods of regions in HMM universe for TF ChIP collection. D) Likelihoods of regions in HMM universe for B-LCL ATAC collection. E) Likelihoods of regions in HMM universe for Random ATAC collection.*

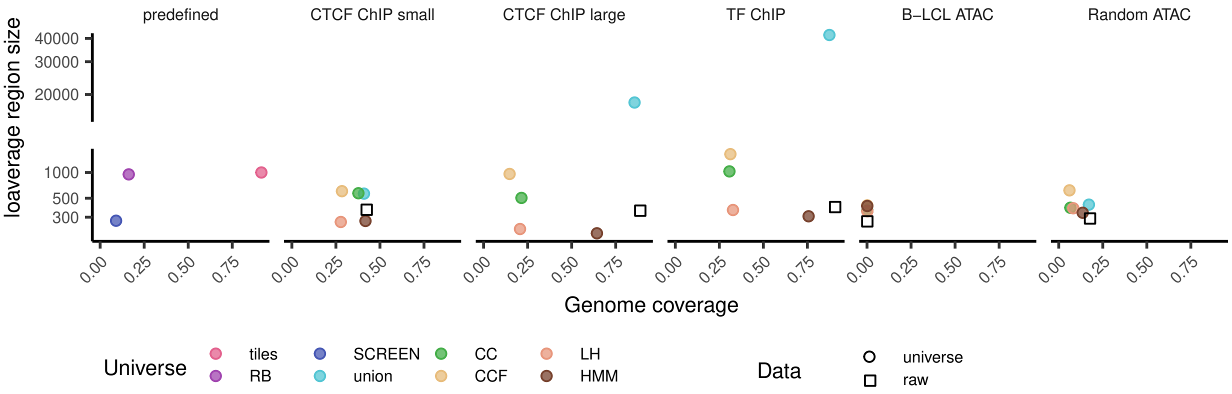

Figure S4: ***Comparison of universe general statistics.*** *Squares denote properties of raw underlying collection.*

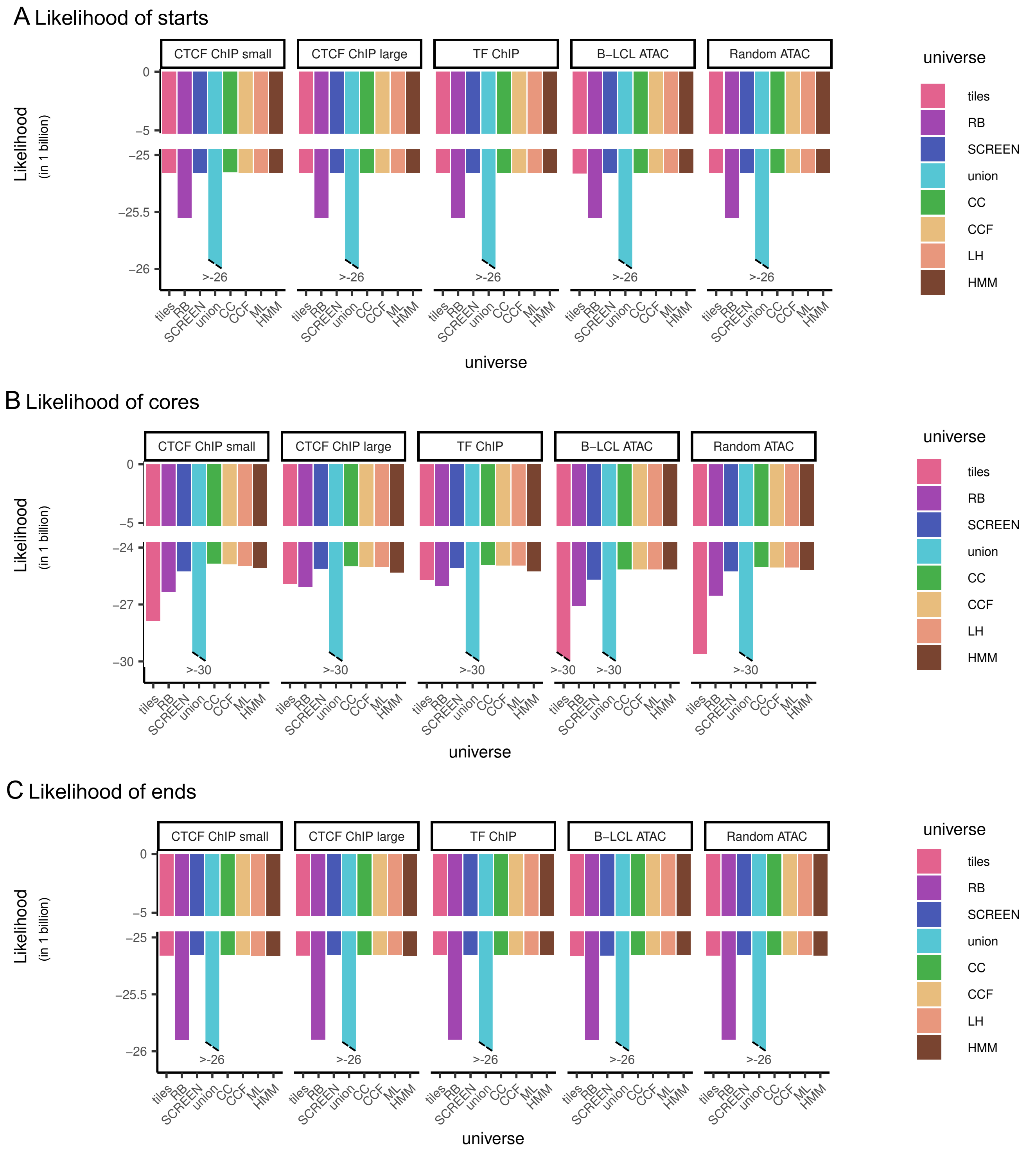

__Figure S5: Likelihood of given parts of region.__ A) Likelihood of region starts by universe and collection. B) Likelihood of region cores by universe and collection. C) Likelihood of region ends by universe and collection.

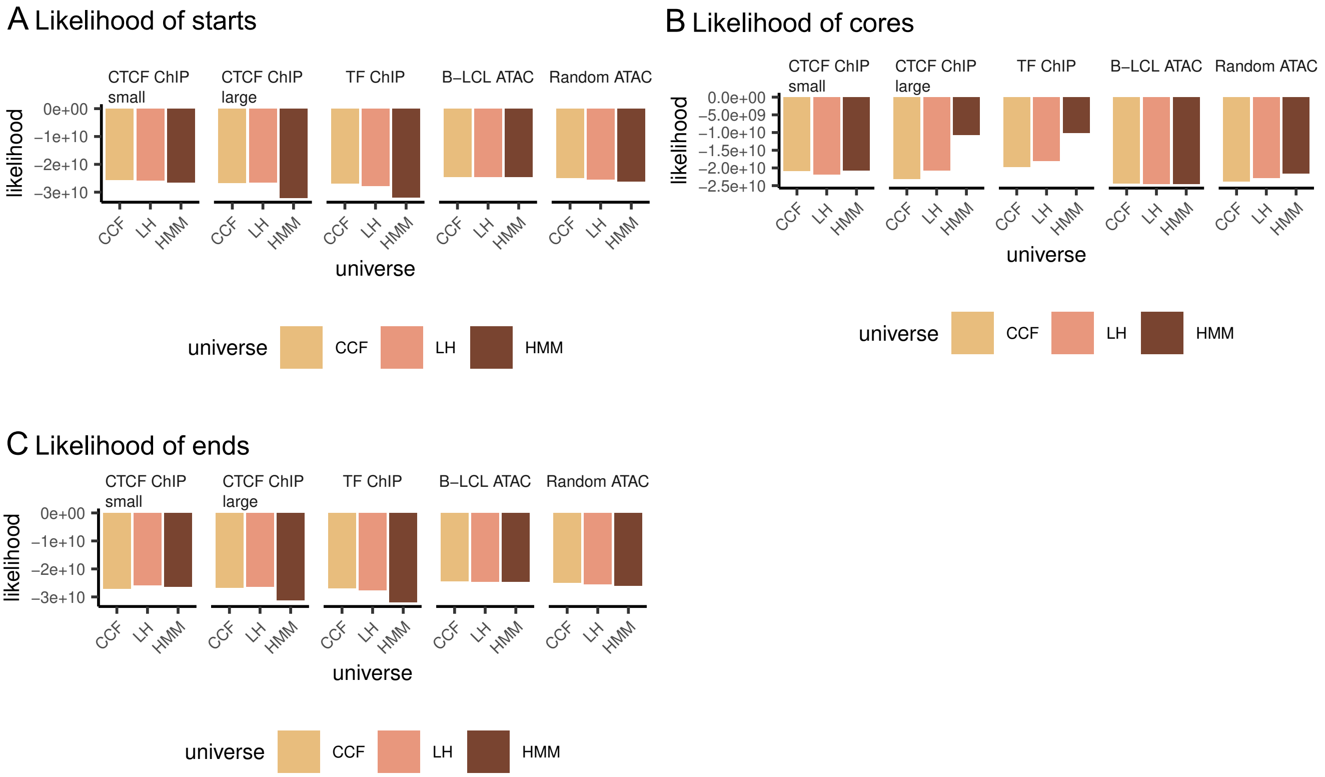

Figure S6*: *Flexible version of likelihood of given parts of region. *A) Flexible likelihood of region starts by universe and collection. B) Flexible likelihood of region cores by universe and collection. C) Flexible likelihood of region ends by universe and collection.*
